# YAP/TAZ Related BioMechano Signal Transduction and Cancer Metastasis

**DOI:** 10.3389/fcell.2019.00199

**Published:** 2019-10-04

**Authors:** Bridget Martinez, Yongchao Yang, Donald Mario Robert Harker, Charles Farrar, Harshini Mukundan, Pulak Nath, David Mascareñas

**Affiliations:** ^1^Engineering Institute, Los Alamos National Laboratory, Los Alamos, NM, United States; ^2^Applied Modern Physics, Los Alamos National Laboratory, Los Alamos, NM, United States; ^3^Department of Medicine, St. George’s University School of Medicine, St. George’s, Grenada; ^4^Chemistry Division, Physical Chemistry and Applied Spectroscopy, Los Alamos National Laboratory, Los Alamos, NM, United States; ^5^Energy and Global Security, Argonne National Laboratory, Lemont, IL, United States

**Keywords:** cancer biology, biomarkers, metastasis and actin dynamics, cell rigidity measurement, cell morphodynamics

## Abstract

Mechanoreciprocity refers to a cell’s ability to maintain tensional homeostasis in response to various types of forces. Physical forces are continually being exerted upon cells of various tissue types, even those considered static, such as the brain. Through mechanoreceptors, cells sense and subsequently respond to these stimuli. These forces and their respective cellular responses are prevalent in regulating everything from embryogenic tissue-specific differentiation, programmed cell death, and disease progression, the last of which being the subject of extensive attention. Abnormal mechanical remodeling of cells can provide clues as to the pathological status of tissues. This becomes particularly important in cancer cells, where cellular stiffness has been recently accepted as a novel biomarker for cancer metastasis. Several studies have also elucidated the importance of cell stiffness in cancer metastasis, with data highlighting that a reversal of tumor stiffness has the capacity to revert the metastatic properties of cancer. In this review, we summarize our current understanding of extracellular matrix (ECM) homeostasis, which plays a prominent role in tissue mechanics. We also describe pathological disruption of the ECM, and the subsequent implications toward cancer and cancer metastasis. In addition, we highlight the most novel approaches toward understanding the mechanisms which generate pathogenic cell stiffness and provide potential new strategies which have the capacity to advance our understanding of one of human-kinds’ most clinically significant medical pathologies. These new strategies include video-based techniques for structural dynamics, which have shown great potential for identifying full-field, high-resolution modal properties, in this case, as a novel application.

## Introduction

Cancer is defined as a set of diseases in which cells bypass the mechanisms that normally limit their growth and replication capacity (Hanahan and Weinberg, 2000; Brower, 2007). This uncontrolled growth is characterized by overexpression of oncogenes, coupled with the loss of tumor suppressor genes (Hanahan and Weinberg, 2000). Eventually, this uncontrolled replication is followed by the invasion of nearby or distant tissue (Hanahan and Weinberg, 2000). Metastasis, or the spreading of a secondary cancer *via* the translocation of cancer cells to different parts of the body, is the cause of over 90% of human cancer deaths (Weigelt et al., 2005; Brower, 2007). This stark percentage highlights the importance of understanding the metastatic processes in cancer and the need to explore and elucidate more effective treatment options. These efforts are currently hindered by many factors, one of them being that a tumor’s microenvironment imparts anti-cancer drug resistance (Lin et al., 2017), making a tumor’s milieu an attractive area of study in the search for novel and unique anti-cancer strategies. Although, several environmental factors have been shown to increase the risk of cancer development (Evanthia et al., 2012; Di Ciaula et al., 2017), it is well known that the human genome plays a crucial role (Thean et al., 2017; Wilson et al., 2017; Gooskens et al., 2018) suggesting that future studies most employ a holistic approach in both the undertaking and subsequent interpretation.

## Microenvironments and Mechanical Cell Signaling

Over the last 30 years, studies have emerged which highlight the effect of mechanical signals on cell behavior (Dupont et al., 2011; Wei et al., 2015; Lachowski et al., 2017). These signals, which emerge from the cells’ microenvironment are influenced by a wide variety of physical forces, including blood flow, gravity, muscle contractions and tissue rigidity (Butcher et al., 2009; Discher et al., 2009; Jaalouk and Lammerding, 2009; Panciera et al., 2017). The prevalence of physical forces, and its implications are observed in a range of fields spanning embryology, physiology as well as pathology. For example, mechanical force has been implicated in directing embryonic development (Farge, 2003; Ruiyi et al., 2006; Page-McCaw et al., 2007; Krieg et al., 2008). Studies show that force is necessary for proper tissue organization. Mesenchymal stem cells differentiate to lineages based on the specific composition of its surrounding matrix (Engler et al., 2006).

## Extracellular Matrix Composition and Cancer Progression

**FIGURE 1:**
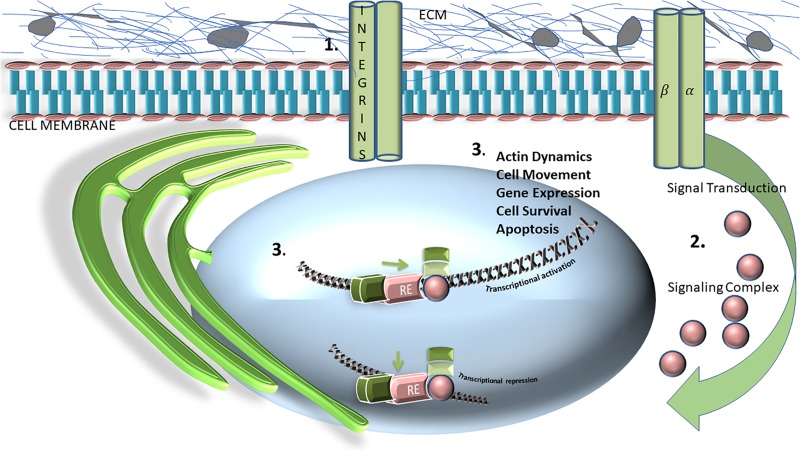
Schematic representation and general overview of extracellular matrix (ECM) matrix and its relationship with transcriptional activation, or repression. Integrins, consisting of, and labeled as alpha and beta subunits, are transmembrane heterodimeric glycoprotein receptors are comprised of an extracellular domain, a transmembrane domain, and a cytoplasmic tail. This schematic represents how the integrin cytoplasmic domains directly associates with various cytoskeletal proteins **(1)** and intracellular signaling molecules **(2)**, which are crucial for modulating fundamental cell processes and functions including cell adhesion, proliferation, migration, and survival **(3)**.

It is clear that the ECM balances forces that maintain tissue homeostasis. The ECM is a dynamic, 3-dimensional, non-cellular structure that is constantly undergoing remodeling in response to mechanical and genetic cues (Bateman et al., 2009; Figure 1). ECM is composed of over 300 different proteins, all in compositional homeostasis, regulating respective structure and function, the precise configuration of which varies with tissue type (Hynes and Naba, 2012). The ECM comprises both the interstitium, as well as the basement membrane, and in this capacity is poised to be in constant interactions with epithelial cells. These interactions enable signal transduction processes which potentially regulate cell health/survival, apoptosis, migration and cell division among many others functions (Hynes, 2009; Hynes and Naba, 2012; Mushtaq et al., 2018; van Helvert et al., 2018). The main structural component of the ECM are collagens- which provide tensile strength and limit distensibility (Hynes and Naba, 2012). Other components include varying types of proteoglycans and glycoproteins, which include elastin (Hynes and Naba, 2012). Additional noteworthy components of the ECM are lysyl oxidase and hydroxylase (LOX and LOXL) (Kirschmann et al., 2002; Payne et al., 2007; Nilsson et al., 2016; Esposito et al., 2018). These secreted copper-dependent amine oxidases function to cross-link collagen and elastin, enabling them to play key roles in tumor progression and metastasis (Nilsson et al., 2016; Esposito et al., 2018). Indeed, many studies have outlined the effects and mechanism of action of LOX/LOXL in a myriad of oncogenic settings (Palamakumbura et al., 2009; Barker et al., 2012; Cox et al., 2016).

The importance of maintenance of ECM biophysical properties can be extrapolated from studies showing that inappropriate alterations are associated with fibrotic conditions and metastatic cancer. Indeed, it is well recognized that the ECM of a tumor is largely distinct from its counterpart in healthy cells (Aszódi et al., 2006; Cox and Erler, 2011; Sotgia et al., 2012; Leight et al., 2017). Studies which outline how post-translational cross-linking of the ECM in premalignant breast cancer is a necessary step for transformation into a malignant cancer suggest that the ECM’s biochemical composition is of paramount importance in cancer progression in this response (Paszek et al., 2005; Levental et al., 2009). This highly impactful data underscores the significance of ECM homeostasis and highlights the necessity to better understand the mechanism(s) that drive ECM remodeling in the context of oncology.

## From Mechanical to Molecular

The topology and rigidity of the extracellular matrix (ECM) is used as a counterbalancing external force against internal pulling forces (Vogel and Sheetz, 2006). Cell structure appears to be regulated by cycles of mechano-sensing, mechano-transduction and mechano-response; where a local sensing of spatial geometry is translated into biochemical cues which can eventually control cell growth, differentiation, shape changes and cell death as previously mentioned (Vogel and Sheetz, 2006). It is through this mechanism that macroscopic cues reach the microscopic scale, regulating cell fate through the modulation of gene expression.

Thus, it is now well established that mechanical cues comprise an integral part of both normal and abnormal physiology, or pathology (Jaalouk and Lammerding, 2009). However, the mechanism(s) by which such cues are translated into molecular signals which subsequently drive increases in gene expression are unclear. Given that cells can perceive their position and shape by interpreting such mechanical cues, it is not surprising that any missteps in the translation of such signals can also lead to abnormalities such as cancer. Understanding the molecular mechanisms involved in this process can thus provide strategies for the identification and treatment of cancer. The focus of this manuscript will be on mechano-transducing transcriptional co-activators, changes in whose expression can potentially mediate the development of cancer.

## Mechano-Transducing Transcriptional Co-Activators in Cancer

Yes-associated protein (YAP) and transcriptional co-activator with PDZ binding motif (TAZ), are oncogenic transcriptional co-regulators which are part of the Hippo pathway, and have recently been identified as mechano-transducing transcriptional co-activators (Panciera et al., 2017). YAP/TAZ have been tightly linked to actin cytoskeleton architecture, promoting cancer drug resistance through actin remodeling; by interacting with transcriptional enhancer factor TEF-1, also known as TEA domain family member (TEAD). These transcription factors are able to translate mechanical cues such as ECM rigidity into genomic transcriptional changes which can then promote processes such as stem cell behavior and regeneration (Kim et al., 2016; Panciera et al., 2017). This translational regulation is mediated *via* Rho GTPase activity, making YAP/TAZ necessary for the ECM stiffness-mediated mesenchymal stem cell differentiation described earlier (Deng et al., 2006; Dupont et al., 2011). Mechanical stimuli and tension create force-dependent mechanical restriction, which results in the inhibition of nuclear pores for molecular transport, thereby directly driving YAP nuclear translocation (Elosegui-Artola et al., 2017). YAP and TAZ are thus re-localized into the nucleus in cells that experience enhanced signaling from processes such as cytoskeletal tension, or increased stretch (Elosegui-Artola et al., 2017), resulting in YAP-dependent and TAZ-dependent biological effects, including cell proliferation (Aragona et al., 2013). In addition, results suggest that the restriction of transport/translocation is further regulated *via* the addition of mechanical stability, a property which the transported protein exhibits (Elosegui-Artola et al., 2017). This determines both the active and passive nuclear transport of YAP, as well as other proteins (Elosegui-Artola et al., 2017). YAP and TAZ are thus necessary for uncontrolled cell proliferation, overcoming contact inhibition, and consequently, are very important in cancer development and metastasis (Kanellos et al., 2015; Zanconato et al., 2016). The microenvironment of tumors is known to impart specific mechanical inputs, which are a consequence of aberrant tissue organization, metalloproteinase-mediated ECM remodeling and ECM stiffening (Tang et al., 2013), which in turn induce YAP and TAZ overactivity in cancer cells. ECM remodeling and stiffening are interesting candidates of study in the understanding of YAP- and TAZ-mediated conversion of benign neoplastic cells into cancer stem cells. Intrinsically, cells appear to be tumor-suppressive, and while remaining in relatively unagitated mechanical conditions, experience low mechanical forces, which leads to low levels of YAP/TAZ. However, in disturbed tissue architecture ECM stiffening induces YAP/TAZ activation/nuclear translocation (Finch-Edmondson and Sudol, 2016; Wang et al., 2016). Recent published studies have validated that active nuclear import indeed controls YAP localization by using AFM as a means for force application on nuclei, results of which suggest that increased active nuclear import is the mediator of YAP nuclear translocation when force is applied both directly as well as indirectly (Elosegui-Artola et al., 2017).

The mechanism linking tissue rigidity and YAP/TAZ-mediated uncontrolled proliferation in tumor cells is not well understood, and offers possible new research targets against epithelial mesenchymal transition (EMT). For example, it is still not clear whether the development of cancer results in mechanical stimuli which induces YAP activity or whether Yap induction contributes to the reorganization, however it is likely that both scenarios are possible, one being driven by genetic predispositions for example, while the other, likely environmental. EMT is a cellular process by which immotile epithelial cells are converted into motile mesenchymal cells via the degradation of the underlying basement membrane. In normal cells, epithelial cells tightly adhere to the basement membrane, which is thus responsible for holding the cells in place (Yang and Weinberg, 2008). The transformation of epithelial cells to motile mesenchymal cells is characterized by the attainment of migratory capacity, invasiveness, resistance to apoptosis and increased ECM production leading to cell rigidity (Kalluri and Weinberg, 2009).

A critical element of EMT is an actin-dependent protrusion of cell pseudopodia; resulting in the destabilization of the actin cytoskeleton, which is a key component of EMT-driven cancer metastasis (Shankar et al., 2010). Known mesenchymal cell markers included FTS binding protein FAP, FSP-1, N-cadherin, vimentin, fibronectin, and β- catenin, among others (Kalluri and Weinberg, 2009; Fernandez-Garcia et al., 2014). Given that YAP is a regulator of these markers, it is not surprising that EMT is promoted by YAP overexpression (Yuan et al., 2016). Recent studies have affirmed that cytoskeleton stability plays an important role in cancer metastasis, given its association with EMT (Sun et al., 2015). Understanding the physical interplay of this intracellular filament network, and its real-time contribution to cancer metastasis is an intriguing therapeutic target against cancer, and the focus of our research. Studies to date have focused on actin cytoskeleton reorganization using live cell imaging and GFP-coronin, as an F-actin reporter (Mun and Jeon, 2012). In a study investigating the relationship of matrix stiffness in EMT, it was found that in genetically engineered pancreatic cancers, fibrotic rigidities promote elements of EMT (Rice et al., 2017). Pancreatic cancer is one of the stiffest of all human solid carcinomas (Rice et al., 2017). Pancreatic ductal adenocarcinoma (PDAC) is an aggressive malignancy characterized by the presence of extensive desmoplasia and myofibroblast-like, high matrix secreting phenotype (Lachowski et al., 2017). Stiffness is also an associated finding in other cancers. For instance, in breast cancer ECM stiffness induces EMT through mechano-transduction, leading to nuclear translocation of YAP and TAZ (Dupont et al., 2011; Wei et al., 2015). In HER2-amplified breast cancer cells, fibronectin, type IV collagen, and matrix rigidity all contribute to drug resistance as tumor microenvironments modulate and dampen the efficacy of targeted anti-cancer therapies (Allen and Louise Jones, 2011; Lin et al., 2017).

## Bridging the Gap – New Methods Toward Understanding the Cellular Biophysics in Cancer

There is a need for more sensitive techniques in order to address the gaps in this field; current research aims are to develop and use sensitive image analyses techniques for quantitatively characterizing structural dynamics at the cellular level- specifically, in cells undergoing cancer metastasis. There still exists significant gaps in quantitatively analyzing the data from current techniques, and in using them to verify and validate finite element models describing the mechanics of cells. This is primarily because of the lack of comprehensive techniques to measure the full-field structural stiffness of cells. With its resolution in the order of fractions of a nanometer, atomic force microscopy (AFM) is one of many methods currently being evaluated in order to obtain high-resolution imaging of cell surface topography as it offers the convenience of being able to measure forces and elasticity in fixed and living cells (Naitoh et al., 2010; Garcia and Proksch, 2013). Applications thus far have included the study of cellular events including locomotion, differentiation and aging, physiological activation and electromotility, as well as cell pathology (Lekka et al., 1999; Nagayama et al., 2001; Müller and Dufrêne, 2011). Bimodal force microscopy uses AFM with several eigenmode excitations frequencies which can be detected, allowing the different resonances to act as channels, which can then be used to isolate material properties such as Young’s modulus of elasticity (a measure of stiffness) (Rodrìguez and Garcìa, 2004; Proksch, 2006). The detection of the cantilever interaction with the sample is monitored and translated into a three-dimensional image (Mozafari et al., 2005), which can reveal heterogeneities of mechanical properties of both the surface and subsurface of cells (Kuznetsova et al., 2007). The methods so far discussed have allowed us to gain preliminary insight into the contribution of mechanical signals and changes in the development and propagation of cancer. However, even with AFM, there are limitations such as the addition of unwanted background flow modes from piezoelectric shakers (Guan et al., 2017). Additionally, AFM sensitivity significantly decreases when probing properties in a liquid medium, given that frequency modulation and dual-frequency imaging are designed for operation in air (Minary-Jolandan et al., 2012). Recently, this limitation has been averted with a unique technique which images cells using AFM with a long-needle probe using only the tip of the probe as the point of interaction with a liquid medium, and spares the cantilever’s high-quality resonant modes by maintaining its air-functioning quality and providing non-contact viscoelastic imaging of living cells (Guan et al., 2017). AFM interrogation of YAP/TAZ has shown that force application to the nucleus is enough to enable YAP translocation independent of rigidity, focal adhesions, the actin cytoskeleton, and also cell-cell adhesion using a constant force of 1.5 nN to the cell nucleus, using cantilevers with 20-μm spherical tips (Elosegui-Artola et al., 2017). In addition, force application to the nucleus increased the nuclear/cytosolic YAP ratio, and then proceeded to return to baseline levels when the force was withdrawn, suggesting that force disrupts YAP distribution and that the observed equilibrium lasts only while force is being applied (Elosegui-Artola et al., 2017). Additionally, it should be noted that these studies have found that there were no measurable differences in YAP ratios when the probe was used to press outside the nucleus, suggesting that the forces produced by exposure to a stiff environment are exerted through focal adhesions and are thereby able to reach the nucleus (Elosegui-Artola et al., 2017). Lastly, because a force- induced breakage of the nucleocytoplasmic barrier cannot be responsible for such an immediate nuclear export, it rules out the possibility that these translocation are simply due to high nuclear deformations (Elosegui-Artola et al., 2017).

Another interesting non-contact/non-intrusive imaging method currently used for diagnostic purposes is interferometric phase microscopy (IPM), which provides several advantages over AFM (Bishitz et al., 2014). The appeal of IPM is that it captures the entire amplitude and phase data from the optical illumination of the sample, generating an optical thickness fluctuation map, which indicates rigidity strength for every cell in the field of view (Bishitz et al., 2014), with the added benefit of cell to cell comparison. However, even as we circumvent existing limitations and find novel techniques that increase imaging sensitivity, it is imperative to note that we are only beginning to understand the relationship between biophysical properties of cells, and their potential to regulate tumorigenesis and motility (Paszek and Weaver, 2004). There is a need for novel and unique imaging techniques develop that provide verification and validation of finite element models of cellular structure.

## Author Contributions

BM devised the project, the main conceptual ideas to be explored, and manuscript outline, worked out the technical details of the review, and performed the literature review and data gathering. All authors contributed substantial context addition, editing and revision of all drafts.

## Conflict of Interest

The authors declare that the research was conducted in the absence of any commercial or financial relationships that could be construed as a potential conflict of interest.
